# Enhanced Walking-Induced Fat Oxidation by New Zealand Blackcurrant Extract Is Body Composition-Dependent in Recreationally Active Adult Females

**DOI:** 10.3390/nu14071475

**Published:** 2022-04-01

**Authors:** Mark E. T. Willems, Milena Banic, Roseanna Cadden, Lara Barnett

**Affiliations:** 1 Institute of Sport, Nursing and Allied Health, College Lane, University of Chichester, Chichester PO19 6PE, UK; milena.banic1990@gmail.com (M.B.); rosiecadden@hotmail.co.uk (R.C.); lara.barnett98@outlook.com (L.B.); 2 Faculty of Health Sciences and Sport, University of Stirling, Stirling FK9 4LA, UK

**Keywords:** anthocyanins, substrate oxidation, exercise, body composition

## Abstract

New Zealand blackcurrant (NZBC) extract enhanced cycling-induced fat oxidation in female endurance athletes. We examined in recreationally active females the effects of NZBC extract on physiological and metabolic responses by moderate-intensity walking and the relationship of fat oxidation changes with focus on body composition parameters. Twelve females (age: 21 ± 2 y, BMI: 23.6 ± 3.1 kg·m^−2^) volunteered. Bioelectrical bioimpedance analysis was used for body composition measurements. Resting metabolic equivalent (1-MET) was 3.31 ± 0.66 mL·kg^−1^·min^−1^. Participants completed an incremental walking test with oxygen uptake measurements to individualize the treadmill walking speed at 5-MET. In a randomized, double-blind, cross-over design, the 30 min morning walks were in the same phase of each participant’s menstrual cycle. No changes by NZBC extract were observed for walking-induced heart rate, minute ventilation, oxygen uptake, and carbon dioxide production. NZBC extract enhanced fat oxidation (10 responders, range: 10–66%). There was a significant correlation for changes in fat oxidation with body mass index; body fat% in legs, arms, and trunk; and a trend with fat oxidation at rest but not with body mass and habitual anthocyanin intake. The NZBC extract responsiveness of walking-induced fat oxidation is body composition-dependent and higher in young-adult females with higher body fat% in legs, arms, and trunk.

## 1. Introduction

The energy requirements of moderate-intensity exercise are provided primarily by the oxidation of carbohydrates and lipids [1]. The relative contribution by the oxidation of carbohydrates and lipids towards the moderate-intensity exercise requirements are affected by dietary intake [2], training status [3], sex [4], exercise modality [5], environmental conditions [6], and supplementation (e.g., caffeine [7], green tea extract [8], Matcha green tea [9], New Zealand blackcurrant [10]). Many studies have examined the factors that contribute to exercise-induced maximal fat oxidation. For example, unfit women with obesity have a higher maximal fat oxidation than unfit women with normal weight [11]. However, observations that body composition may affect exercise-induced fat oxidation are not consistent. Kerhervé et al. [12] observed that body composition differences between women did not affect exercise-induced fat oxidation but noted substantial inter-individual differences in normal weight, overweight, and obese women. In addition, fat mass localization may affect exercise-induced fat oxidation [13]. However, studies on the effect of supplementation-induced fat oxidation have not addressed the potential effect of regional fat distribution. Women with similar body mass and body mass index may still vary substantially in the localization of body fat. In general, enhanced fat oxidation by supplementation is considered beneficial with implications for individuals with weight-management issues. For example, Moro orange extract seems to have lipolytic effects and reduces abdominal fat [14]. Although a substantial reduction of energy intake is most effective for weight loss (e.g., [15]), dietary choices may be helpful for weight management. In addition, for the general population, there is an interest to affect the exercise-induced metabolic responses to get enhanced health benefits. Many supplements can stimulate lipolysis [16]. Enhanced fat oxidation by supplement use can be partly due to enhanced lipolysis with the overall metabolic effect decided by the summed effects of regional lipolysis. In Şahin et al. [17], effects of New Zealand blackcurrant extract on whole-body fat oxidation by moderate-intensity walking was affected by body composition in recreationally active males. In males, higher body mass index and body fat% provided higher levels of enhanced whole-body exercise-induced fat oxidation [17]. Whether such observations can be transferred to women is not known. In addition, in Şahin et al. [17], the relationship for regional body fat% with enhanced fat oxidation was not examined. Therefore, the main aim of the present is to examine the effects of intake of New Zealand blackcurrant extract on physiological and metabolic responses by moderate-intensity treadmill walking in recreationally active women. In addition, we will primarily examine whether there is a relationship between the blackcurrant-induced changes in whole-body fat oxidation and body fat% in upper limb, lower limbs, and trunk.

## 2. Materials and Methods

### 2.1. Participants

The female participants (*n* = 12, age: 21 ± 2 years; height: 166 ± 7 cm; body mass: 65 ± 11 kg; BMI: 23.7 ± 3.1 kg·m^−2^) recruited for the study were healthy, recreationally active Caucasian university students and staff. All females had a regular menstrual period, were non-smokers, and were not taking other dietary supplements. Participants provided written informed consent, and health status was confirmed by a health history questionnaire. The physical activity level of the female participant’s physical activity level was quantified with the short version of the International Physical Activity Questionnaire (2749 ± 2106 MET·week^−1^) [18]. The present study accepted the following methods of contraception: combined pill, diaphragm, or intrauterine device. Approval for the study was obtained from the University of Chichester Research Ethics Committee (ethical approval code: 1819_1600944).

### 2.2. Experimental Design

The study had a randomized, placebo-controlled, cross-over design. Female participants visited the laboratory for one preliminary and two experimental sessions. For each session, participants abstained from unaccustomed and intense exercise for 48 h, had no alcohol and caffeine intake for 24 h, and were at least 2 h postprandial after consuming a breakfast of one slice of bread and a glass of water [10]. Sessions were in the morning with a month between the two experimental sessions to ensure that the females were tested in the same phase of the participant’s menstrual cycle [19]. The participants were supplemented with placebo or NZBC extract (see below for details) for seven days before each experimental session [10,20]. Participants completed a food frequency questionnaire with anthocyanin-containing foods and drinks listed in the Phenol-Explorer database [21] to estimate daily habitual anthocyanin intake (32 ± 26 mg·day^−1^; range: 5 to 88 mg·day^−1^).

### 2.3. Preliminary Session

In the preliminary session, height (Seca 213, Seca, Birmingham, UK) and body mass (Kern ITB, Kern, Germany) were measured as well as body composition (Tanita BC418, segmental body composition analyzer, Tanita, IL, USA). Subsequently, participants were fitted with a heart-rate monitor (Polar F1, Polar Electro (UK) Ltd., Warwick, UK). Participants then rested for 30 min in a chair, followed by 2 × 10 min expired air collection using Douglas bags (Cranlea & Co. Bourneville, Birmingham, UK) to determine the oxygen consumption at rest. The lowest value for oxygen consumption at rest was taken as the one metabolic equivalent (1-MET) (1-MET: 3.31 ± 0.66 mL·kg^−1^·min^−1^). Heart rate at rest was 69 ± 9 beats·min^−1^. Blood pressure at rest was taken twice (OMRON 705 IT, Medisave, Weymouth, UK) and averaged (systolic blood pressure: 120 ± 8 mmHg, diastolic blood pressure: 70 ± 7 mmHg). Subsequently, participants completed an incremental-intensity walking protocol on a treadmill (Woodway Ergo ELG 70, Cranlea & Co. Bourneville, Birmingham, UK). Treadmill incline was 1%. Participants completed 5 × 8-min stages starting at 2 km∙h^−1^, progressing by 1 km∙h^−1^ until a speed of 6 km∙h^−1^ was reached [22]. In the last 3 min of each 8 min stage, expired air was collected using Douglas bags. Expired air was analyzed for fractions of oxygen and carbon dioxide by a 3-point calibrated gas analyser (Series 1400, Servomex, Crowborough, East Sussex, UK), and volume was measured (Harvard Apparatus Ltd., Edenbridge, UK). Expired gas volumes were corrected to standard temperature and pressure and dry gas conditions and calculated using Haldane transformation with consideration of inspired fractions of oxygen and carbon dioxide that were measured each time halfway during each 3 min expired-air collection. The incremental-intensity walking protocol with measurement of oxygen consumption was performed to establish for each participant the linear relationship between walking speed and the metabolic equivalent (r^2^ = 0.9626 ± 0.0259). The linear relationship between walking speed and metabolic equivalent allowed for each participant to establish the walking speed at 5-METs (i.e., moderate-intensity exercise, walking speed: 5.53 ± 0.39 km∙h^−1^).

### 2.4. Supplementation for the Experimental Sessions

For the experimental sessions, two capsules of NZBC extract (one capsule containing 300 mg active cassis, of which 105 mg were anthocyanins, i.e., 35–50% delphinidin-3-*O*-rutinoside, 5–20% delphinidin-3-*O*-glucoside, 30–45% cyanidin-3-*O*-rutinoside, 3–10% cyanidin-3-*O*-glucoside) (CurraNZ, Health Currancy Ltd., Surrey, UK) or placebo pills (2 × 300 mg microcrystalline cellulose M102) were taken for 7 days. The final two capsules were taken 2 h before visiting the laboratory for the experimental sessions. Participants were allowed a breakfast consisting of one slice of bread and a glass of water 3 h before visiting for the experimental sessions.

### 2.5. Experimental Sessions

For the experimental sessions, participants completed a 30 min moderate-intensity treadmill walk (4.7 ± 0.4 METs) at the speed established in the preliminary session with recording of heart rate (Polar F1, Polar Electro (UK) Ltd., Warwick, UK) and expired-air collection from 7–10, 17–20, and 27–30 min. Rates of whole-body fat and carbohydrate oxidation were calculated with equations below from Jeukendrup and Wallis [23] and the assumption of negligible protein oxidation:

$$\mathrm{Fat}\;\mathrm{oxidation}\;\left(g\times \min^{-1}\right)=1.695\times \;\dot{V}O_{2}-1.701\times \;\dot{V}{\mathrm{CO}}_{2}$$

$$\mathrm{Carbohydrate}\;\mathrm{oxidation}\;\left(g\times \min^{-1}\right)=4.210\times \;\dot{V}{\mathrm{CO}}_{2}-2.962\times \;\dot{V}O_{2}$$

The respiratory exchange ratio was calculated by dividing the volume of carbon dioxide produced by the volume of oxygen consumed.

### 2.6. Statistical Analysis

Statistical analyses were completed using Graphpad Prism 5 for Windows (Graphpad Software, San Diego, CA, USA). Physiological and metabolic responses by the moderate-intensity walk were measured from 7–10, 17–20, and 27–30 min during the walk and averaged. All parameters were tested for normality with the D’Agostino and Pearson omnibus test. Two-tailed paired sample *t*-tests were used to compare all the parameters between the placebo and NZBC extract conditions. Values are reported as mean ± SD and 95% confidence intervals. Statistical significance was accepted at *p* < 0.05. *p*-Values of 0.05 ≥ *p* ≤ 0.1 were interpreted according to guidelines by Curran-Everett and Benos [24]. Cohen’s d effect sizes were calculated and considered trivial (*d* < 0.2), small (*d* = 0.2–0.49), moderate (*d* = 0.5–0.79), and large (*d* ≥ 0.8). Pearson correlation coefficients were calculated and tested for significance for the relationships between habitual anthocyanin intake (not considering the NZBC extract intake); fat oxidation at rest; body mass; body mass index; fat% of the legs, arms, and trunk; and changes of walking-induced fat oxidation with intake of NZBC extract in comparison to walking-induced fat oxidation in the placebo condition. The ∆ FAO is the fat oxidation with intake of New Zealand blackcurrant extract minus the fat oxidation with intake of placebo. The required sample size was not calculated, and the number of 12 participants was lower than previous studies with observations of an effect of NZBC extract on exercise-induced fat oxidation (males, *n* = 14 [10]; males, *n* = 15 [17,25]; females, *n* = 16 [26]).

## 3. Results

### 3.1. Walking-Induced Physiological Responses

During the moderate-intensity treadmill walk, the NZBC extract had no effect on heart rate (PL: 121 ± 17 beats·min^−1^, 95% CI (110, 132 beats·min^−1^); NZBC extract: 120 ± 14 beats·min^−1^, 95% CI (111, 129 beats·min^−1^); *p* = 0.58, *d* = −0.06), minute ventilation (PL: 25.8 ± 7.4 L·min^−1^, 95% CI (21.1, 30.5 L·min^−1^); NZBC extract: 24.4 ± 6.3 L·min^−1^, 95% CI (20.5, 28.4 L·min^−1^), *p* = 0.14, *d* = −0.20), oxygen uptake (PL: 16.1 ± 2.1 mL·kg^−1^·min^−1^, 95% CI (14.8, 17.5 mL·kg^−1^·min^−1)^; NZBC extract: 15.8 ± 1.8 mL·kg^−1^·min^−1^, 95% CI (14.6, 16.9 mL·kg^−1^·min^−1^), *p* = 0.45, *d* = −0.18), and carbon dioxide production (PL: 13.8 ± 2.1 mL·kg^−1^·min^−1^, 95% CI (12.5, 15.1 mL·kg^−1^·min^−1^); NZBC extract: 13.1 ± 1.4 mL·kg^−1^·min^−1^, 95% CI (12.2, 14.0 mL·kg^−1^·min^−1^), *p* = 0.17, *d* = −0.40). The absence of an effect on the physiological responses by NZBC extract indicate no change in the regulatory mechanism for exercise-induced heart rate and respiratory demands.

### 3.2. Walking-Induced Metabolic Responses

**Figure 1:**
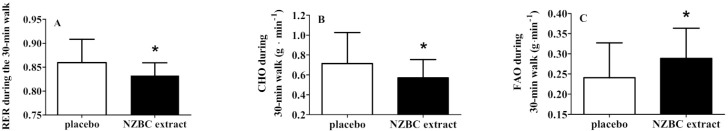
(**A**) Respiratory exchange ratio, (**B**) carbohydrate oxidation, and (**C**) fat oxidation during 30-min of moderate-intensity treadmill walking. Data are mean ± SD from 12 female participants. NZBC, New Zealand blackcurrant; RER, respiratory exchange ratio; CHO, carbohydrate oxidation; FAO, fat oxidation; *, indicates a difference with the placebo condition (*p* < 0.05).

New Zealand blackcurrant extract provided 3.0% lower values for RER (PL: 95% CI (0.83, 0.89), NZBC extract: 95% CI (0.82, 0.85), *p* = 0.009, *d* = −0.69) (Figure 1A), 10.8% lower values for carbohydrate oxidation (PL: 95% CI (0.51, 0.91 g·min^−1^), NZBC: 95% CI (0.45, 0.69 g·min^−1)^, *p* = 0.03, *d* = −0.56) (Figure 1B), and 25.0% higher values for fat oxidation (PL: 95% CI (0.19, 0.30 g·min^−1^), NZBC extract: 95% CI (0.24, 0.34 g·min^−1^), *p* = 0.005, *d* = 0.59) (Figure 1C). The 10 participants (~83%) with increased walking-induced fat oxidation responded by an average of 32% (SD: 17%, range: 10–66%), with 9 participants higher than 14%. The changes in metabolic responses by moderate-intensity walking with intake of NZBC extract indicate a change in the regulation of exercise-induced carbohydrate and fat metabolism.

### 3.3. Habitual Anthcoyanin Intake, Fat Oxidation at Rest, and Walking-Induced Fat Oxidation

**Figure 2:**
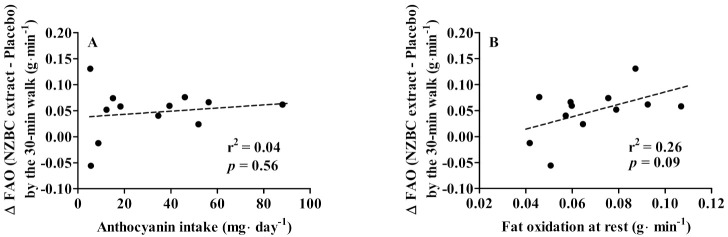
Relationship between habitual anthocyanin intake (**A**) and fat oxidation at rest (**B**) and the changes in fat oxidation (∆ FAO) by 30 min of moderate-intensity treadmill walking with intake of New Zealand blackcurrant (NZBC) extract. The ∆ FAO is the fat oxidation with intake of New Zealand blackcurrant extract minus the fat oxidation with intake of placebo. The habitual anthocyanin intake did not include the anthocyanin intake by New Zealand blackcurrant extract. The dotted lines are the linear regression lines.

There was no significant correlation between habitual dietary anthocyanin intake and the absolute change in walking-induced whole-body fat oxidation (r^2^ = 0.04, *p* = 0.56) (Figure 2A). The habitual dietary intake of anthocyanins did not include the anthocyanins consumed by intake of the NZBC extract. The absence of a relationship between habitual dietary intake and the absolute change in exercise-induced fat oxidation suggests that the enhanced metabolic response by intake of NZBC extract was not due to a low habitual anthocyanin intake. There was a trend for a significant correlation between fat oxidation at rest and the absolute change in walking-induced fat oxidation (r^2^ = 0.26, *p* = 0.09) (Figure 2B). This suggests an enhanced response for exercise-induced fat oxidation with intake of NZBC extract for females with higher whole-body fat oxidation at rest.

### 3.4. Body Mass, Body Composition, and Walking-Induced Fat Oxidation

**Figure 3:**
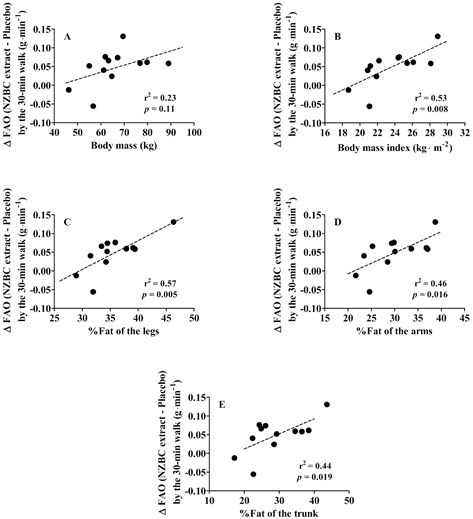
Relationship between body mass (**A**), body mass index (**B**), %fat of the legs (**C**), %fat of the arms (**D**), and %fat of the trunk (**E**) and the changes in fat oxidation (∆ FAO) by 30 min of moderate-intensity treadmill walking with intake of New Zealand blackcurrant (NZBC) extract. The ∆ FAO is the fat oxidation with intake of New Zealand blackcurrant extract minus the fat oxidation with intake of placebo. The dotted lines are the linear regression lines.

There was no significant correlation between body mass and the absolute change in walking-induced fat oxidation (r^2^ = 0.23, *p* = 0.11) (Figure 3A). However, there was a significant correlation between body mass index (r^2^ = 0.53, *p* = 0.008) (Figure 3B), body fat% of the legs (r^2^ = 0.57, *p* = 0.005) (Figure 3C), body fat% of the arms (r^2^ = 0.46, *p* = 0.016) (Figure 3D), and body fat% of the trunk (r^2^ = 0.44, *p* = 0.019) (Figure 3E) and the absolute change in whole-body fat oxidation.

The observations on body composition parameters suggest an enhanced response for exercise-induced fat oxidation with intake of NZBC extract for females with higher body fat in the legs, arms, and trunk. The significant correlations for the relationship between body mass index; body fat% in the legs, arms, and trunk; and changes in walking-induced fat oxidation may suggest differences in adipocyte sensitivity in response to intake of NZBC extract in females with higher body fat%.

## 4. Discussion

The present study presents novel findings on the effect of intake of anthocyanin-rich New Zealand blackcurrant extract on the physiological and metabolic responses during moderate-intensity walking in recreationally active females. Recently, Elliott-Sale et al. [27] emphasized the need for inclusion of women as participants in exercise science studies. Our findings contribute to the limited information that is available on the ergogenic effects during exercise by intake of a berry supplement in studies with only female participants. Previous studies have reported on the enhanced exercise-induced fat oxidation by intake of New Zealand blackcurrant extract (7 days, 210 mg anthocyanins per day) in recreationally active males during moderate-intensity walking [20] and endurance-trained females during 2 h of cycling at 65% $\;\dot{V}$O_2max_ [26]. In the study by Strauss et al. [26], no information was provided on body composition parameters of the female participants. In Şahin et al. [17] (14 days intake, 210 mg anthocyanins per day), enhanced walking-induced fat oxidation was higher with overall body fat% in males. In the present study, we examined whether the enhanced fat oxidation in females was related to body mass; body mass index; body fat% in legs, arms, and trunk; habitual anthocyanin intake; and baseline fat oxidation in rest. The main findings of the present were (1) substantial enhanced walking-induced fat oxidation with intake of New Zealand blackcurrant extract in recreationally active females, and (2) the enhanced walking-induced fat oxidation with intake of New Zealand blackcurrant in recreationally active females was significantly correlated with body mass index and body fat% in legs, arms, and trunk. The present study used a dosing strategy of 7 days’ intake with exercise modality and intensity similar to Şahin et al. [20]. Şahin et al. [20] reported enhanced walking-induced fat oxidation of with intake of New Zealand blackcurrant extract of 11% in adult males (age: 26 ± 6 years, body fat%: 15 ± 5%), whereas the present study reports for adult females (body fat%: 31 ± 6%) an increase by 25%. Interestingly, endurance-trained males in Cook et al. [25] and endurance-trained females in Strauss et al. [26] had similar dosing strategies (7 days’ intake with 210 mg of anthocyanins per day) and provided enhanced 2 h cycling-induced fat oxidation by 21.5% (males) and 27% (females), respectively. Therefore, it seems that females are more responsive to New Zealand blackcurrant extract to enhance exercise-induced fat oxidation, and it is also independent of training status. The complexity and the numerous steps involved to alter exercise-induced fat oxidation and the absence of any biochemical, molecular, and structural markers in the present study limits the interpretation of the substantial effects on walking-induced fat oxidation by intake of New Zealand blackcurrant extract by females. However, it is possible that body composition differences between males and females contributes to the observed effects in the study by Şahin et al. [20] and the present study. Future studies should examine the effects of intake of New Zealand blackcurrant extract on exercise-induced fat oxidation in men and women with similar body fat% although it is recognized that the recruitment for such studies will be challenging. Observations that body composition may affect exercise-induced fat oxidation are not consistent. Kerhervé et al. [12] observed that body composition differences between women did not affect exercise-induced fat oxidation but noted substantial inter-individual differences in normal weight, overweight, and obese women. In the present study, the correlation (and significance) was higher for the relationship between body fat% of the legs and the changes in walking-induced fat oxidation. We speculate that there may be heterogenous adipocyte sensitivity linked with body fat location for the effect of anthocyanin-induced metabolites. If that is the case, adipocytes in the legs may contribute more to the enhanced exercise-induced fat oxidation than adipocytes in the arms and trunk. This may have consequences for the changes in body composition by long-duration intake of anthocyanin-rich blackcurrant supplementation during an exercise intervention. Interestingly, Isacco et al. [13] observed in normal weight, pre-menopausal women that exercise-induced fat oxidation was higher in women with a low abdominal to lower body fat mass ratio. In the present study, we were not able to quantify abdominal fat mass. Future studies should address the effect of long-duration intake of anthocyanin-rich blackcurrant supplementation on body composition.

Robinson et al. [28] reported in recreationally active Caucasian males (*n* = 57, BMI: 24.2 ± 2.6 kg·m^2^) that resting fat oxidation correlated (R = 0.55) with exercise-induced maximal fat oxidation. In addition, in the same study, $\;\dot{V}$O_2max_ correlated (R = 0.44) with resting fat oxidation [28]. In the present study, we showed a trend for the correlation between resting fat oxidation and the change in walking-induced fat oxidation with intake of New Zealand blackcurrant extract, potentially due to the fact that our study only had 12 female participants. Nevertheless, future work may want to address whether in recreationally active males and females the $\;\dot{V}$O_2max_ and thus baseline cardiovascular fitness may be predictive of the response to enhanced walking-induced fat oxidation with intake of New Zealand blackcurrant extract.

The present study used a convenience sample of recreationally active female participants as the primary aim was to examine the effect of intake of New Zealand blackcurrant extract on walking-induced fat oxidation. In a previous study from our group, Matcha green tea drinks in females enhanced walking-induced fat oxidation by 18% [22]. It is possible that the mechanisms for enhanced exercise-induced fat oxidation by different supplementations, i.e., Matcha green tea and New Zealand blackcurrant extract, are not similar. The Matcha green tea study by Willems et al. [22] also had recreationally active females as participants and a follow-up study, with the measurement in another laboratory confirming even higher enhanced fat oxidation of 35% by three weeks’ Matcha intake [9]. Future studies should examine the combined intake of supplementations of which single use has been shown to enhance exercise-induced fat oxidation. Such information may be useful to inform nutritional strategies for individuals with weight-management issues. In addition, the effect of supplementation of fat oxidation during rest as well as longer intake duration in females is also important, and future work is recommended.

A limitation of the present study was that participants did not record a 48 h food diary for the two visits with taking placebo or New Zealand blackcurrant extract. However, although previous studies [20,26] had dietary intake recorded, it is unclear how the nutritional components may have interacted with the intake of blackcurrant anthocyanins, potentially affecting the bioavailability of anthocyanin-derived metabolites. It is the anthocyanin-derived metabolites that are potentially linked with adaptative cellular mechanisms.

## 5. Conclusions

In conclusion, in recreationally active adult females, 7-day intake of NZBC extract substantially enhanced fat oxidation by 30 min of moderate-intensity walking exercise. The enhanced walking-induced fat oxidation by intake of NZBC extract in recreationally active females was body composition-dependent.

## Data Availability

The data in this article will be provided by a reasonable request.

## References

[1] Romijn J.A., Coyle E.F., Sidossis L.S., Rosenblatt J., Wolfe R.R. (2000). Substrate metabolism during different exercise intensities in endurance-trained women. J. Appl. Physiol..

[2] Lambert E.V., Speechly D.P., Dennis S.C., Noakes T.D. (1994). Enhanced endurance in trained cyclists during moderate intensity exercise following 2 weeks adaptation to a high fat diet. Eur. J. Appl. Physiol..

[3] Coggan A.R., Kohrt W.M., Spina R.J., Bier D.M., Holloszy J.O. (1990). Endurance training decreases plasma glucose turnover and oxidation during moderate-intensity exercise in men. J. Appl. Physiol..

[4] Devries M.M., Hamadeh M.J., Phillips S.M., Tarnapolsky M.A. (2006). Menstrual cycle phase and sex influence muscle glycogen utilization and glucose turnover during moderate-intensity endurance exercise. Am. J. Physiol. Regul. Integr. Comp. Physiol..

[5] Chenevière X., Malatesta D., Gojanovi B., Borrani F. (2010). Differences in whole-body fat oxidation kinetics between cycling and running. Eur. J. Appl. Physiol..

[6] Maunder E., Plews D.J., Merien F., Kilding A.E. (2020). Exercise intensity regulates the effect of heat stress on substrate oxidation rates during exercise. Eur. J. Sport Sci..

[7] Ruiz-Moreno C., Gutiérrez-Hellín J., Amaro-Gahete F.J., González-García J., Giráldez-Costas V., Pérez- García V., Del Coso J. (2021). Caffeine increases whole-body fat oxidation during 1 h of cycling at Fatmax. J. Nutr..

[8] Venables M.C., Hulston C.J., Cox H.R., Jeukendrup A.E. (2008). Green tea extract ingestion, fat oxidation, and glucose tolerance in healthy humans. Am. J. Clin. Nutr..

[9] Willems M.E.T., Fry H.L., Belding M.A., Kaviani M. (2021). Three Weeks Daily Intake of Match Green Tea Powder Affects Substrate Oxidation during Moderate-Intensity Exercise in Females. J. Diet. Suppl..

[10] Cook M.D., Myers S.D., Blacker S.D., Willems M.E.T. (2015). New Zealand blackcurrant extract improves cycling performance and fat oxidation in cyclists. Eur. J. Appl. Physiol..

[11] Frandsen J., Hansen I.M.D., Wismann J.F., Olsen M.H., Brage-Andersen M.R., Sahl R.E., Hansen M., Ingersen A., Modvig J.L., Schmücker M. (2021). Maximal Fat Oxidation Rate is Higher in Fit Women and Unfit Women with Obesity, Compared to Normal-weight Unfit Women. J. Clin. Endocrinol. Metabol..

[12] Kerhervé H.A., Harvey L.M., Eagles A.N., McLellan C., Lovell D. (2020). Similar rates of fat oxidation during graded submaximal exercise in women of different body composition. PLoS ONE.

[13] Isacco L., Ennequin G., Boisseau N. (2020). Effect of Fat Mass Localization on Fat Oxidation during Endurance Exercise in Women. Front. Physiol..

[14] De Lima L.P., de Paula Barbosa A. (2021). A review of the lipolytic effects and the reduction of abdominal fat from bioactive compounds and moro orange extracts. Heliyon.

[15] Nackers L.M., Middleton K.R., Dubyak P.J., Daniels M.J., Anton S.D., Perri M.G. (2013). Effects of prescribing 1,000 versus 1,500 kilocalories per day in the behavioral treatment of obesity: A randomized trial. Obesity.

[16] Kim J., Park J., Lim K. (2016). Nutrition Supplements to Stimulate Lipolysis: A Review in Relation to Endurance Exercise Capacity. J. Nutr. Sci. Vitaminol..

[17] Şahin M.A., Bilgiç P., Montanari S., Willems M.E.T. (2022). Daily and not Every-Other-Day Intake of Anthocyanin-Rich New Zealand Blackcurrant Extract Alters Substrate Oxidation during Moderate-Intensity Walking in Adult Males. J. Diet. Suppl..

[18] Lee P.H., Macfarlane D.J., Lam T.H., Stewart S.M. (2011). Validity of the International Physical Activity Questionnaire Short form (IPAQ-SF): A systematic review. Int. J. Behav. Nutr. Phys. Act..

[19] Bonen A., Haynes F.J., Watson-Wright W., Sopper M.M., Pierce G.N., Low M.P., Graham T.E. (1983). Effects of menstrual cycle on metabolic responses to exercise. J. Appl. Physiol. Respir. Environ. Exerc. Physiol..

[20] Şahin M.A., Bilgiç P., Montanari S., Willems M.E.T. (2021). Intake Duration of Anthocyanin-Rich New Zealand Blackcurrant Extract Affects Metabolic Responses during Moderate Intensity Walking Exercise in Adult Males. J. Diet. Suppl..

[21] Neveu V., Perez-Jiménez J., Vos F., Crespy V., du Chaffaut L., Mennen L., Knox C., Eisner R., Cruz J., Wishart D. (2010). Phenol-Explorer: An online comprehensive database on polyphenol contents in foods. Database.

[22] Willems M.E.T., Şahin M.A., Cook M.D. (2018). Matcha Green Tea Drinks Enhance Fat Oxidation during Brisk Walking in Females. Int. J. Sport Nutr. Exerc. Metab..

[23] Jeukendrup A.E., Wallis G.A. (2005). Measurement of substrate oxidation during exercise by means of gas exchange measurements. Int. J. Sports Med..

[24] Curran-Everett D., Benos D.J. (2004). Guidelines for reporting statistics in journals by the American Physiological Society. Adv. Physiol. Educ..

[25] Cook M.D., Myers S.D., Gault M.L., Edwards V.C., Willems M.E.T. (2017). Dose effects of New Zealand blackcurrant on substrate oxidation and physiological responses during prolonged cycling. Eur. J. Appl. Physiol..

[26] Strauss J.A., Willems M.E.T., Shepherd S.O. (2018). New Zealand blackcurrant extract enhances fat oxidation during prolonged cycling in endurance-trained females. Eur. J. Appl. Physiol..

[27] Elliott-Sale K.J., Minahan C.L., de Jonge X.A.K.J., Ackerman K.E., Sipilä S., Constantini N.W., Lebrun C.M., Hackney A.C. (2021). Methodological Considerations for Studies in Sport and Exercise Science with Women as Participants: A Working Guide for Standards of Practice for Research on Women. Sports Med..

[28] Robinson S.L., Chambers E.S., Fletcher G., Wallis G.A. (2016). Lipolytic Markers, Insulin and Resting Fat Oxidation are Associated with Maximal Fat Oxidation. Int. J. Sports Med..

